# Is the Concept of Avian Pathogenic *Escherichia coli* as a Single Pathotype Fundamentally Flawed?

**DOI:** 10.3389/fvets.2014.00005

**Published:** 2014-10-14

**Authors:** Charlotte Collingwood, Kirsty Kemmett, Nicola Williams, Paul Wigley

**Affiliations:** ^1^Department of Infection Biology, Institute of Infection and Global Health, School of Veterinary Science, University of Liverpool, Neston, UK; ^2^Department Epidemiology and Population Health, Institute of Infection and Global Health, School of Veterinary Science, University of Liverpool, Neston, UK

**Keywords:** chicken, *Escherichia coli*, APEC, broiler chicken, eggs

## Abstract

Avian Pathogenic *Escherichia coli* (APEC) is a major pathogen within the poultry industry. However disease, especially in broiler chickens, may be caused by range of *E. coli* genotypes that carry few, if any, virulence factors associated with APEC. Furthermore, commensal *E. coli* in the intestines of healthy birds may carry an array of APEC virulence factors suggesting they have potential to cause disease when opportunity arises. Given the diseases caused by APEC, namely colibacillosis and salpingitis peritonitis syndrome, are syndromic in nature and the great diversity of the strains causing disease we suggest it is wrong to consider disease as the result of a single APEC pathotype. Whilst it is clear certain pathogenic *E. coli* can be considered as APEC, much of the disease-associated with *E. coli* in domestic poultry is as much a consequence of increased host susceptibility due to stress, immunosuppression, co-infection, or poor welfare. This leads to more “opportunistic” infections rather than the result of infection with a specific pathotype. As such the current use of the term APEC for all cases of *E. coli* infection in the chicken is fundamentally flawed.

Avian Pathogenic *Escherichia coli* (APEC) is both a primary and secondary pathogen of the chicken and other avian species (1). It is considered to be a member of the extra-intestinal pathogenic *E. coli* (ExPEC) along with human Uropathogenic (UPEC) and neonatal meningitis-associated *E. coli* (NMEC) that cause disease outside the intestine. APEC infection may occur in broiler (meat) chicken, turkey, and egg-laying sectors. In all sectors, infection is syndromic in nature. In the broiler chicken, APEC infections are considered to typically lead to colibacillosis; a syndrome that includes respiratory tract infection, air sacculitis, pericarditis, perihepatitis, splenomegaly, and swollen head syndrome. In mature laying hens, reproductive tract infection leading to salpingitis or salpingo-peritonitis syndrome (SPS) is common.

Avian pathogenic *Escherichia coli* is amongst the greatest health threats to the developed poultry industries and its emergence perhaps reflects the decrease in the prophylactic use of antimicrobials or their use as growth promoting agents. Furthermore, the close genetic relationship between APEC and other ExPEC associated with human disease along with evidence from experimental animal models have lead to suggestions that APEC may represent a zoonotic risk (2–8).

Whilst in recent years, APEC has become accepted as a primary pathogen rather than a consequence of respiratory or immunosuppressive viral infections, our understanding of APEC and its pathogenesis has remained relatively limited, due, at least in part, to its great diversity and genomic plasticity (3, 4, 9–13). This variation is true of other ExPECs too (14). *E. coli* associated with human intestinal disease harbor certain defining virulence factors such as Type III secretion systems, Shiga-like toxins, and enterotoxins. ExPEC are, in general, less well defined in terms of their virulence determinants and are more variable between isolates. These virulence determinants include bacterial capsule that helps avoid host innate immunity, adhesins such as fimbriae involved in attachment to host cells and tissues, hemolysins that lyse host blood cells and multiple systems for acquisition of iron needed by the bacterium. In APEC, no single common virulence factor has been identified in all strains. Although certain genes associated with pathogenicity are common in APEC, including: *iss* associated with serum resistance (15), *ibeA* associated with invasion (16), and *sitA* associated with iron acquisition, they are not found in all isolates. Virulence genes including *iss*, *iroN*, and the *iuc* and *cva* operons are often associated with large plasmids including the Colicin V (ColV) plasmid (11). Certain *E. coli* serotypes such as O1 and O78 are more frequently associated with colibacillosis. Potentially these lineages harbor the genetic backbone required to acquire virulence mechanisms. Representatives of these serotypes have been genome sequenced and characterized (17, 18) and whilst there is little doubt these represent highly pathogenic variants of APEC, the reality is that in commercial broiler chickens colibacillosis is caused by a wide range of *E. coli* serotypes. Recently in our laboratory we have, for the first time, compared *E. coli* isolates causing colibacillosis within a broiler flock with those carried as intestinal commensals (19). These data showed that many colibacillosis – associated isolates carry few, if any, of the genes most commonly ascribed as APEC virulence factors. In essence, they have the genotype of “intestinal commensals.” Equally, virulence genes may be found in “commensal” *E. coli* residing in the intestines of presumed healthy birds. Furthermore, isolates from cases of colibacillosis are not associated with any specific phylogenetic group. It appears that soon after hatch chicks acquire a diverse range of *E. coli* as part of their microbiota, likely to be sourced from the hatchery environment. Within this population are isolates carrying virulence-associated genes (1), though the frequency of such genes decreases as birds age (19). *E. coli* found in the intestinal tract are likely to form a reservoir of potential infection, we have previously termed such isolates as potential APEC or pAPEC (19), and may be associated with early infection and mortality. Such isolates could be considered opportunists in a compromised avian host as a consequence of production-related stress, immunosuppression, or prior infection (1, 19, 20). Whilst opportunistic infections are likely to reduce in likelihood in older birds, they represent a clear risk in broiler production.

In laying hens, the situation is a little clearer. We have previously shown that APEC isolates from an outbreak of SPS in a commercial layer flock was caused by a single isolate that displayed features of both APEC and UPEC (2). Full genomic analysis of this isolate is ongoing. Recently, we have looked at the distribution of key virulence in genes in 188 SPS isolates from the UK through PCR. These findings suggest the majority possess *iss*, *hlyF* (a hemolysin), and the iron acquisition genes *iucC* and *iroN* but other virulence genes are less frequently found (Figure 1). Whilst within the 188 isolates there is variation, there does at least seem to be at least greater commonality of virulence factors than in colibacillosis, although seven of these isolates do not posses any of the genes screened for. This closer relationship between certain genotypes and disease and layers has also been described in Denmark where it appears certain lineages are common throughout the country, though there is still considerable diversity (21). Intriguingly in our UK-based studies although genes of the *iuc* aerobactin operon are found commonly (over 70%) in both SPS isolates and in systemically isolated *E. coli* in colibacillosis, *iss* which was found in 83% SPS isolates was only found in 25% of broiler colibacillosis isolates whilst *ibe*, was found in more than 60% of broiler isolates but was found in <25% of layer SPS isolates. This may be coincidence, but does suggest that different virulence factors may play roles causing what are very different diseases. The use of the subcutaneous infection model for cellulitis in day old chicks has recently identified *pic*, a serine protease, as a putative a virulence factor (22). However as yet, this is only an association with reduced virulence, has no mechanism ascribed and has not been identified in other models. A problem is that phenotypes of virulence factors in the chicken are poorly understood, partly as a consequence of the difficulty in reproducing experimental infections, in particular of the reproductive tract, so making our understanding of the mechanisms of APEC disease rather rudimentary. Recently developed infection models for SPS may clarify some of the virulence factors and mechanisms that underlie this disease syndrome (23, 24). However, such models rely on direct delivery to the reproductive tract and, in common with colibacillosis models, where delivery is direct to the air sacs (18, 25), may fail to detect important factors involved in the initiation of infection and colonization of tissues.

**Figure 1:**
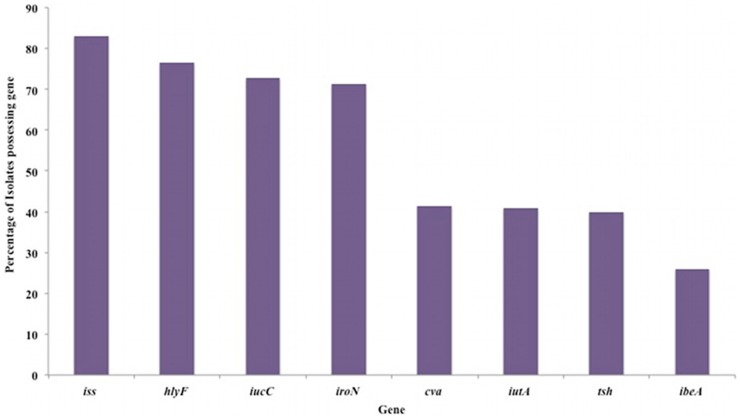
**Distribution of APEC associated virulence determinants in 188 *E. coli* isolates from cases of salpingitis peritonitis syndrome in UK laying hens**. Virulence determinants detected by PCR using previously described methods (13, 19). The genes tested and their function were *iss* (increased serum survival), *hlyF* (a hemolysin*), iucC* (aerobactin), *iroN* (iron acquisition), *cva* (colicin operon), *iutA* (iron transport), *tsh* (*a hemagglutinin*), and *ibeA* (invasion). Data previously presented at American Society for Microbiology, Annual General Meeting, Denver, June 2013.

So one may pose the question should APEC be defined as any *E. coli* isolated from a diseased or sick bird or should the term be more narrowly defined? It is clear that certain well-characterized isolates can be defined as APEC. They can cause disease in experimental models and possess a range of virulence factors. However disease, and in particular colibacillosis, may result from infection with an isolate which bears few or none of the hallmarks by which we would define APEC, other than the fact it has caused disease. The use of the term APEC for *E. coli* that cause what in all likelihood is opportunistic infection in the chicken is fundamentally flawed. It is perhaps more appropriate to consider both colibacillosis and SPS as disease syndromes caused by an array of variable genotypes, that are as dependent on host susceptibility as any virulence factor possessed by the pathogen. It is probably fair to say APEC is successful pathogens that are far more likely to lead to disease in poultry than commensal *E. coli*. However, disease is not just restricted to those isolates we can define as having an APEC pathotype, and trying to pin all incidences of colibacillosis or SPS on an APEC pathotype is flawed. Of course such concepts are more challenging both to scientists and producers when identifying specific causative agents and their mechanisms are the norm.

There are also implications in disease control. It is difficult to control bacteria that are normally a commensal and although vaccination against APEC (26), especially in laying hens, has value it is not feasible to achieve either economically or immunologically in young broiler chicks. As such, good hatchery hygiene and management are important in controlling early mortalities, with good management and welfare likely to reduce the risk of colibacillosis in growing broilers. This also includes effective control of other pathogens including respiratory viruses where *E. coli* is a common secondary pathogen. Vaccination may not be effective against such a diverse microbial population and removal of *E. coli* as part of the microbiota may have other implications we cannot foresee. That said, approaches to sequence multiple genomes of *E. coli* from the chicken could reveal a common “core” genome in disease-associated *E. coli*, identifying a genetic relationship that cannot be found when considering virulence factors alone. Such an approach may also lead to the identification of novel targets for future vaccines.

In conclusion, we believe that *E. coli* disease in the chicken cannot be simply defined as being caused by a single pathotype of *E. coli*. In particular, colibacillosis is perhaps better defined as disease caused by *E. coli* rather than by Avian Pathogenic *E. coli*, and that the term APEC be reserved for the smaller number of well-defined “bona fide” pathogenic isolates with a range of defined virulence determinants that can reproduce disease in animal models. There are APEC, but not all disease-associated with *E. coli* in the chicken is caused by APEC.

## Conflict of Interest Statement

The authors declare that the research was conducted in the absence of any commercial or financial relationships that could be construed as a potential conflict of interest.
